# Mpox-HIV co-infection in a low-incidence setting: epidemiological insights from Qinghai, China

**DOI:** 10.3389/fpubh.2026.1764643

**Published:** 2026-06-05

**Authors:** Jiaying Kuang, Yongkai Shi, Shicun Dong, Xiaojing Li, Hailan Cao, Da Liang, Zhijian Tang, Fangming Guo, Ning Fan, Peng Guo, Pengfei Zhao, Lili Xu, Wei Li

**Affiliations:** 1 Qinghai Provincial Center for Disease Control and Prevention, Xining, Qinghai, China; 2 Xining Center for Disease Control and Prevention, Xining, Qinghai, China; 3 The Fourth People's Hospital of Qinghai Province, Xining, Qinghai, China

**Keywords:** co-infection, epidemiological, HIV, mpox, Qinghai province

## Abstract

**Objective:**

Using the first reported case of mpox and HIV co-infection in Qinghai Province, China, as a case study, this paper examines the epidemiological characteristics and transmission dynamics of mpox–HIV co-infection in low-incidence settings, as well as systemic gaps in surveillance, diagnosis, and response. It aims to generate broadly applicable insights for mpox prevention and control in similar low-incidence regions worldwide.

**Methods:**

A retrospective epidemiological investigation was conducted involving Case A, a patient with mpox–HIV co-infection reported by a hospital in Qinghai Province on 24 June 2025, and the patient’s close contacts. Case and close-contact definitions were applied in accordance with World Health Organization (WHO) criteria, the *Chinese Guidelines for the Diagnosis and Treatment of Mpox* (2022 Edition), and the *Chinese Technical Guidelines for Mpox Prevention and Control* (2022 Edition). Face-to-face, on-site epidemiological investigations were conducted to collect and analyse the patient’s epidemiological history, clinical diagnosis, treatment information, and laboratory test results. All biological samples were collected by specialist laboratory personnel during the on-site epidemiological investigation of the mpox case, in strict accordance with national standard testing methods and laboratory quality-control protocols. Both the patient and close contacts signed informed consent forms, and the research process was conducted in accordance with the relevant principles of the Declaration of Helsinki.

**Results:**

Case A was a male patient living with HIV who reported sex with men. He first developed perineal pruritus on 19 June. The following day, his temperature rose to 39.4 °C, and his symptoms did not improve after medication. He sought medical attention at a hospital on 23 June and was discharged on 7 July after full recovery; the total duration of illness was 18 days. During the 21 days before symptom onset, the patient had no history of international or out-of-city travel, no history of smallpox vaccination, and no history of contact with wild animals. He had, however, recently experienced high-risk exposure within the men who have sex with men (MSM) community. On 24 June, the admitting hospital collected a nasopharyngeal swab specimen for mpox virus (MPXV) nucleic acid testing, which confirmed a diagnosis of mpox. On 27 June, genetic sequencing was performed on a venous blood specimen from the patient, and the virus was identified as MPXV clade IIb. The sequence was further assigned to the C.1.1 sublineage, full designation B.1.3.1.1, within MPXV clade IIb, formerly known as the West African clade. A total of three close contacts were identified, all of whom tested negative for MPXV nucleic acid. One close contact tested positive for MPXV IgG antibodies, suggesting previous exposure to MPXV. Taken together, the epidemiological history, serological findings, and timing of symptom onset suggest that high-risk sexual contact was the most likely route of exposure for Case A. The onset of symptoms was consistent with the typical mpox incubation period of 7–14 days after the reported high-risk sexual exposure. Phylogenetic analysis showed that the sequence most closely related to that from Case A was EPI_ISL_19893388, submitted by Taiwan, China. Previously reported cases in Qinghai Province were predominantly imported. However, Case A had no clear history of international travel or travel outside the province and therefore did not meet the typical epidemiological profile of an imported case. The transmission pattern was therefore more consistent with local transmission within specific social or sexual networks. This finding suggests that silent transmission may already be occurring in low-incidence regions.

**Discussion:**

Through a retrospective analysis of the first reported case of mpox–HIV co-infection in Qinghai Province, this study identified three key findings. First, the findings provide evidence of previously unrecognized exposure and possible silent transmission in a low-incidence region. These results may inform the early identification of hidden community transmission and the strengthening of early warning systems. The mpox virus sequence most closely related to that from Case A had been submitted from Taiwan, China. None of the three identified close contacts presented with typical clinical symptoms, such as fever or rash; however, one tested positive for MPXV IgG antibodies. Because all close contacts had high-risk exposure to Case A, this finding suggests the possibility of prior, unrecognized exposure within the local high-risk population network associated with the case. However, serological evidence alone cannot establish the exact source, timing, or direction of transmission. Second, this study shows that mpox surveillance in low-incidence areas relies primarily on passive reporting by healthcare institutions and lacks active screening mechanisms for high-risk populations. The effectiveness of passive reporting depends directly on individuals’ willingness to seek medical care and their ability to recognize symptoms. Case A sought medical attention and was diagnosed only after the onset of a typical rash; earlier symptoms, such as fever and fatigue, did not receive sufficient attention, and the patient did not seek timely care. This suggests that individuals with mild, atypical, or asymptomatic infections may be missed by surveillance systems if they do not proactively seek medical attention. These findings provide evidence to support the optimization of active surveillance models in low-incidence areas and the transition from passive surveillance to more proactive prevention and control strategies. Third, this study highlights an important serological gap in mpox epidemiology and emphasizes the value of serological testing in identifying previous exposure and reconstructing possible transmission chains. Some infected individuals may not present with a typical rash or may have only mild symptoms. Moreover, after viral nucleic acid becomes undetectable, IgM and IgG antibodies may persist for an extended period. Serological markers can therefore help identify previous exposure, asymptomatic infection, and silent transmission, thereby addressing surveillance blind spots associated with reliance on case reports and nucleic acid testing alone. In summary, mpox prevention and control in low-incidence areas should priorities the risk of silent transmission. This requires strengthening proactive screening among high-risk groups, reducing over-reliance on healthcare-seeking behavior, and establishing dynamic serological surveillance systems to improve the early identification and control of silent transmission.

## 1 Introduction

The global mpox outbreak in 2022 marked the virus’s expansion beyond its historically endemic regions, transforming mpox from a localized disease in parts of Africa into a global public health challenge, with significant shifts in transmission dynamics, clinical characteristics and control strategies (1). Unlike previous outbreaks, the global epidemic has been characterized by marked clustering of mpox transmission, with rapid spread particularly among higher-risk groups such as men who have sex with men and individuals with multiple sexual partners. Because these groups are also at increased risk of human immunodeficiency virus (HIV) infection, the epidemiological association between mpox and HIV has become increasingly prominent and has emerged as a central focus of global public health concern (2–5). HIV infection can impair immune function, potentially increasing susceptibility to mpox virus infection, exacerbating clinical symptoms, prolonging disease course and increasing the risk of severe illness and death. Conversely, the spread of mpox may also heighten the risk of HIV transmission, creating a bidirectional epidemiological dynamic that further increases the complexity and difficulty of epidemic control (6, 7).

Although global systems for the surveillance and diagnosis of mpox have gradually been established, the global spread of the virus has made shortcomings and gaps in these systems increasingly apparent, particularly in low-incidence regions. To date, most global research on mpox has focused on the prevention and control of cluster outbreaks in high-incidence areas (8), whereas insufficient attention has been paid to the operational effectiveness of surveillance and diagnostic systems in low-incidence regions; this remains a significant research gap. As a low-incidence region for mpox in China, Qinghai Province has previously reported mainly imported cases, and local transmission has been rare. Consequently, its mpox surveillance and response system has not yet been comprehensively tested against an outbreak of local transmission, making Qinghai a useful setting for stress-testing mpox prevention and control capabilities in low-incidence regions. This study examines the first reported case of mpox–HIV co-infection at a hospital in Qinghai. Through a retrospective epidemiological investigation and analysis of laboratory testing data, it focuses on potential silent transmission associated with mpox–HIV co-infection in low-incidence regions, the dependence of surveillance efforts on healthcare-seeking behavior and serological gaps in mpox epidemiology. This study not only fills a gap in case-based research in Qinghai Province and provides a reference for the prevention and control of mpox–HIV co-infection in the province, but also offers valuable epidemiological insights for other low-incidence regions worldwide in addressing these issues and refining their prevention and control systems.

## 2 Materials and methods

### 2.1 Study design

This study used a retrospective epidemiological investigation design, with Case A, the first reported case of mpox–HIV co-infection reported by a hospital in Qinghai on 24 June 2025, as the primary subject of investigation. All close contacts of this case were also included in the investigation. The definitions of Case A and close contacts were applied in accordance with the criteria set out by the World Health Organization (WHO), the *Chinese Guidelines for the Diagnosis and Treatment of Mpox* (2022 Edition), and the *Chinese Technical Guidelines for Mpox Prevention and Control* (2022 Edition). Face-to-face, on-site epidemiological investigations were conducted to collect and analyse the epidemiological history, clinical diagnosis, treatment information, and laboratory test results of Case A and the close contacts, as applicable. Laboratory tests included mpox virus nucleic acid testing, confirmatory HIV antibody testing, HIV viral load testing, CD4^+^ T-lymphocyte counts, and mpox IgG antibody testing. All biological samples were collected by professional laboratory staff during on-site epidemiological investigations of the mpox case and close contacts, in strict accordance with national standard testing methods and laboratory quality-control protocols. This study was a retrospective analysis and did not involve prospective interventions; all data were derived from previously completed clinical and epidemiological investigations. Informed consent was obtained from Case A and all close contacts, and the study was conducted in accordance with the relevant principles of the Declaration of Helsinki.

### 2.2 Definitions

With reference to the official definition of the World Health Organization (9), China’s *Guidelines for the Diagnosis and Treatment of Mpox* (2022 Edition) (10), and China’s *Technical Guidelines for the Prevention and Control of Mpox* (2022 Edition) (11), the following definitions were applied.

1. A confirmed case of mpox was defined as a person presenting with an acute rash of unknown aetiology accompanied by fever or lymphadenopathy who, within 21 days before symptom onset, had a history of any of the following epidemiological exposures: travel to regions with reported mpox cases; contact with a confirmed or suspected case of mpox; same-sex sexual activity; or a sexual partner who engaged in same-sex sexual activity. A confirmed case was also required to have tested positive for mpox virus nucleic acid or to have had mpox virus isolated in laboratory testing.
2. A close contact was defined as a person with any of the following exposures occurring between 7 days before symptom onset in the case and the time when all rashes had completely crusted over: direct skin contact with the case’s rash, scabs, or body fluids; sexual or intimate contact, including mouth-to-mouth, mouth-to-skin, or skin-to-skin intimate contact; contact with contaminated items, including clothing, bedding, or objects contaminated by the case; close-range droplet or aerosol exposure, defined as prolonged face-to-face contact within 1 metre for ≥15 min; or occupational exposure, including healthcare or laboratory personnel handling case specimens or contaminated materials without protective equipment.

### 2.3 Data sources and collection

#### 2.3.1 Epidemiological data

Professional public health officers conducted face-to-face, on-site epidemiological investigations of the case and close contacts in accordance with the requirements of the *Technical Guidelines for Mpox Prevention and Control* (2022 Edition), using standardized questionnaires to collect information. The collected information primarily included general demographic information on the case, epidemiological history, time of onset, time of first consultation, sequence and progression of symptoms, and the identification and contact details of close contacts.

#### 2.3.2 Clinical data

Clinical data were sourced from the electronic medical record system and paper-based medical records of a hospital in Qinghai, including admission records, progress notes, medical orders, test reports, diagnostic certificates, and discharge summaries. The extracted information covered clinical symptoms and signs, the clinical diagnostic process, treatment plans, treatment outcomes, and indicators related to prognosis.

#### 2.3.3 Laboratory testing data

Laboratory testing data were provided by specialized laboratories with appropriate testing qualifications. All biological samples were collected and processed by laboratory personnel in accordance with biosafety protocols during the on-site epidemiological investigation of the mpox case. Nasal swabs, vesicular fluid from the left upper limb, venous blood, and perineal scab samples were collected from Case A, whilst throat swabs were collected from close contacts for mpox virus nucleic acid testing. Testing was conducted by the laboratory of the Qinghai Provincial Centre for Disease Control and Prevention using real-time polymerase chain reaction (real-time PCR) and a commercially available mpox-specific fluorescent quantitative PCR kit. A cycle threshold (Ct) value of ≤40 was considered positive.

In addition, the Qinghai Provincial Centre for Disease Control and Prevention conducted whole-genome sequencing of mpox virus from vesicular fluid samples collected from Case A; these samples were sent to the National Institute for Viral Disease Control and Prevention under the Chinese Centre for Disease Control and Prevention for further whole-genome sequencing. Blood samples collected from close contacts were tested using ELISA for mpox virus IgM and IgG antibodies and HIV antibodies. All testing strictly adhered to national standard testing methods and laboratory quality-control protocols, with test results documented in formal reports issued by the laboratory.

## 3 Results

### 3.1 Clinical features

Case A was a 42-year-old unemployed man with HIV infection who reported sexual activity with male partners. In the 21 days preceding symptom onset, he denied a history of travel abroad or to other cities, smallpox vaccination, or exposure with wild animals.

On 19 June, the patient reported itching in the perineal region. The following day, his temperature rose to 39.4 °C and did not improve after taking medication. On 23 June, he sought medical attention at a hospital, where he was admitted to the Department of Integrated Traditional Chinese and Western Medicine with a diagnosis of HIV infection for 8 years with fever. Upon admission, he presented with fever, lymphadenopathy, and vesicular lesions of varying sizes with crusting in the external genital region. A pustular lesion the size of a mung bean was observed on the left upper limb, with no other significant abnormalities noted. His medical history was as follows: HIV-positivity; CD4 + cell count >800 cells/μL; viral load below the detection limit (no viral replication); well-controlled AIDS disease, and no other underlying conditions. Anti-inflammatory and antiviral therapy with ceftriaxone and ganciclovir was administered. After 15 days of treatment, the patient’s vital signs were stable, and his body temperature was normal. No jaundice of the skin or mucous membranes, as well as no subcutaneous hemorrhage, nodules, or scarring were noted. The patient was discharged on 7 July.

### 3.2 Epidemiological network

**Figure 1:**
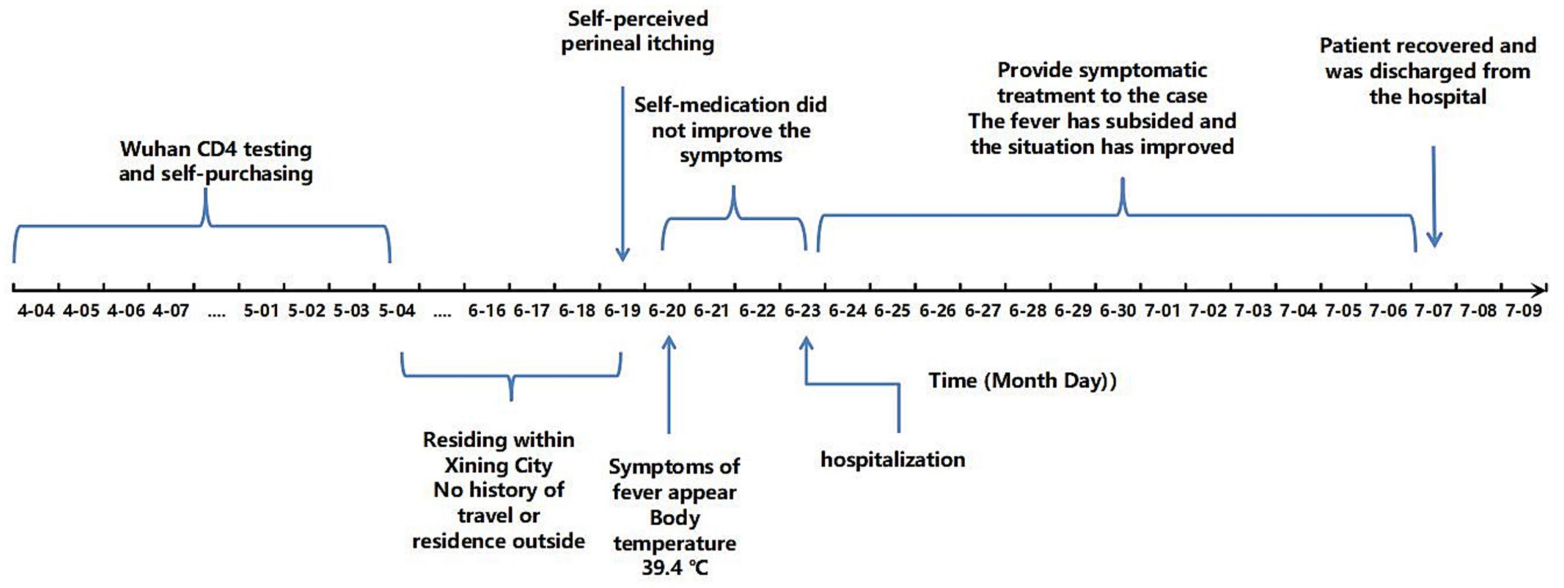
Timeline of symptom onset, medical consultation, and outcome of Case A: the first case of mpox co-infected with HIV in Qinghai province, China.

**Figure 2:**
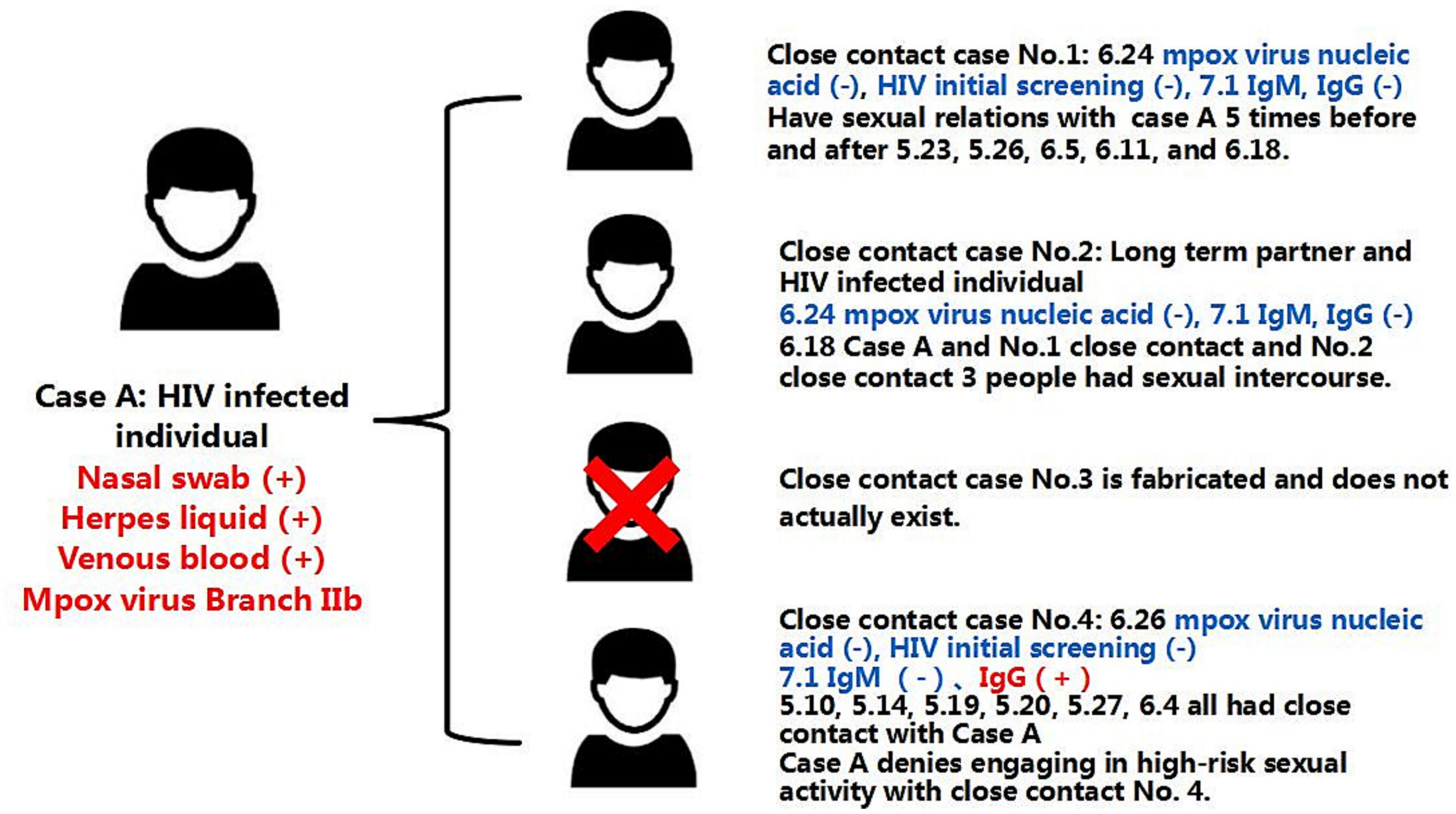
Screening of close contacts of Case A: the first case of mpox co-infected with HIV in Qinghai province, China.

Case A, a long-term resident of Xining City, was diagnosed with HIV infection 8 years prior to this presentation. He confessed to travelling regularly to City A for CD4 testing and to purchase medication; his most recent trip was on April 4. He returned to Xining on May 4, more than 21 days since symptom onset. Case A identified three close contacts (Close Contacts 1, 2, and 3). Close Contact 1, a casual sexual partner of Case A, reported engaging in high-risk sexual activity at the residence of Case A on May 23 and 26, and on June 5, 11, and 18. On June 18, Case A, Close Contact 1, and Close Contact 2 engaged in high-risk sexual activity. Close Contact 2, a long-term sexual partner of Case A, reported having been HIV-positive for 8 years, suffering from rectal cancer, and having had no sexual activity in the prior month. Contact details for Close Contact 3 could not be obtained. Subsequently, through the collaborative epidemiological investigation mechanism of the “Three Publics (Public Health, Public Security, Public Works),” a comparison of movement trajectories confirmed that Close Contact 3 was a fictitious character fabricated by Case A and identified a new close contact (Close Contact 4). As Case A refused to disclose contact history with Close Contact 4, big data from the Ministry of Industry and Information Technology were utilized to retrieve the movement records of Close Contact 4 from May 1 to June 4. Comparison with the records of Case A revealed that between May 1 and 5, Close Contact 4 had left City B to travel to another province. Close contact 4 had close contact with Case A on May 10, 14, 19, 20, and 27 May, as well as on June 4. However, Case A denied having engaged in sexual activity with Close contact 4 (a male). The timeline of onset, medical history, and outcome of Case A are detailed in Figure 1. The investigation findings regarding the close contacts are detailed in Figure 2.

### 3.3 Laboratory test results

#### 3.3.1 Laboratory test results for Case A

On June 24, the Xining Centre for Disease Control and Prevention, in collaboration with the Provincial Centre for Disease Control and Prevention, conducted nucleic acid testing for mpox virus on samples isolated from a nasal swab, fluid from a blister on the left upper limb, venous blood, and a scab from the perineal region of Case A. Both the nasal swab and the blister fluid samples tested positive for mpox virus. On June 27, the laboratory at the Provincial Centre for Disease Control and Prevention conducted mpox virus gene sequencing on the venous blood sample, confirming that it belonged to the mpox IIb lineage. On August 15, the samples were sent to the National Institute for Viral Disease Control and Prevention (NIVDCP) for whole-genome sequence alignment; the results indicated that the virus belongs to the C.1.1 (full name B.1.3.1.1) sublineage of the mpox virus IIb lineage (West African lineage). In the GISAID mpox virus database, the sequence that was most closely related to the virus from Case A is EPI_ISL_19893388, which was submitted by Taiwan, China (sampling date: 8 August 2024).

#### 3.3.2 Laboratory test results for close contacts

Samples from Close Contacts 1, 2, and 4 were tested for mpox virus nucleic acid, HIV antibodies, and mpox antibodies. Close Contacts 1 and 2 tested negative for both mpox virus nucleic acid and antibodies. While Close Contact 4 tested negative for mpox virus nucleic acid, when examined using the HIV screening test, they tested positive for mpox virus serum IgG antibodies, suggesting a risk of prior exposure to the mpox virus. Additional details are provided in Table 1.

**Table 1:** Summary of mpox virus and HIV testing items and results for close contacts.

Close contact	Sample type	Test item	Collection date	Test results
No. 1	Throat swab, blood	Mpox virus nucleic acid detection	2025.06.24	(−)
No. 1	Whole blood	HIV initial screening test	2025.06.24	(−)
No. 1	Serum	Mpox virus antibody IgM and IgG detection	2025.07.01	IgM (−), IgG (−)
No. 2	Throat swab	Mpox virus nucleic acid detection	2025.06.24	(−)
No. 2	Serum	Mpox virus antibody IgM and IgG detection	2025.07.01	IgM (−), IgG (−)
No. 4	Throat swab	Mpox virus nucleic acid detection	2025.06.26	(−)
No. 4	Whole blood	HIV initial screening test	2025.06.26	(−)
No. 4	Serum	Mpox virus antibody IgM and IgG detection	2025.07.1	IgM (−), IgG (+)

### 3.4 Inference on transmission

Based on the epidemiological history of the case in this study, as well as the laboratory test results, close contact testing, and mpox virus genome sequencing results, a cautious inference regarding the source of transmission in this case of concurrent mpox and HIV infection is as follows:

First, the case reported a history of multiple high-risk sexual encounters in the 21 days prior to symptom onset, tested positive for mpox nucleic acid, and was confirmed as an mpox case.

Second, the timing of symptom onset aligned with the mpox incubation period (7–14 days) following high-risk sexual activity. Moreover, genome sequencing revealed that the sequence most closely related to the patient’s infection was submitted from Taiwan, China. None of the three identified close contacts developed mpox-related symptoms. However, one close contact (Close Contact 4) tested positive for mpox IgG antibodies and was identified as a person who had high-risk contact with Case A. This finding suggests that the Taiwanese viral lineage caused silent (asymptomatic) or undetected exposure within the local high-risk population network associated with the case, indicating undiscovered local transmission chains.

Third, the epidemiological evidence argues against recent important findings. Specifically, while previously reported cases in Qinghai Province were predominantly imported, Case A had no history of overseas travel or outside the province in the 21 days prior to symptom onset and no history of contact with confirmed or suspected mpox cases. Furthermore, as no other mpox cases were reported in Qinghai Province during the same period, the possibility of this case being a recent direct imported infection can be excluded. Instead, the source of infection is more consistent with local transmission occurring within certain surveillance networks, suggesting that the risk of silent transmission already exists in regions with low incidence rates.

## 4 Discussion

In terms of geographical distribution, global outbreaks of mpox–HIV co-infection are primarily concentrated in Africa, Europe, and the Americas. Domestically, cases in China have mainly been reported in southern coastal provinces, exhibiting a pattern of localized clusters (1). Although local transmission chains have been identified in some cases, existing global research has largely focused on cluster outbreaks in high-incidence regions. Discussion regarding latent transmission, surveillance limitations, and serological applications in low-incidence regions is extremely scarce. Notably, the present study addresses this research gap and provides a new epidemiological perspective for mpox prevention and control in low-incidence regions worldwide. The present case involved a man who frequently engaged in sexual activity with other men and presented with m-pox–HIV co-infection, along with preserved CD4^+^ T-cell counts intact and no severe clinical complications. This presentation is consistent with global epidemiological patterns: while cases of m-pox–HIV co-infection predominantly occur in populations engaging in high-risk sexual behavior, the risk of severe disease is lower in HIV-infected individuals with mpox co-infection whose immune function is not severely compromised (12). This pattern suggests that the clinical severity of mpox-HIV co-infection is associated with CD4^+^ T-lymphocyte count levels and may have broader clinical relevance.

The characteristics of the cases presented in this study suggest that silent transmission occurs in low-incidence areas. While silent transmission represents a major challenge for mpox prevention and control in low-incidence areas, it remains understudied despite its importance for public health practice (13). Unlike in high-incidence areas, silent transmission in low-incidence areas does not exhibit the typical characteristics of cluster outbreaks; it often occurs sporadically and covertly, with infections primarily occurring within local networks of high-risk populations (14). The absence of typical clinical manifestations allows these cases to evade passive surveillance systems and may ultimately lead to localized cluster outbreaks.

The current mpox surveillance system possesses several limitations. First, it heavily relies on patient-initiated healthcare seeking behavior and passive reporting by hospitals. In the present study, cases only sought medical attention and were diagnosed after the onset of a typical rash. Early symptoms, such as fever and fatigue, received insufficient medical attention, as the patient did not seek timely medical care. This observation suggests that individuals with mild, atypical, or asymptomatic infections are highly likely to evade detection by the surveillance system, as they do not proactively seek medical care. Second, mpox surveillance in low-incidence areas lacks active screening mechanisms for high-risk populations, relying primarily on passive reporting by healthcare facilities. The effectiveness of passive reporting depends directly on the willingness of the reporter to seek medical care and their ability to recognize symptoms, creating substantial gaps in case identification (15–17). Moreover, as mpox cases are rare in low-incidence areas, healthcare workers cannot often recognize the early symptoms of the disease, which further exacerbates the underreporting of cases driven by reliance on the willingness of patients to seek medical care, preventing the surveillance system from promptly detecting early and silent transmission and substantially diminishing its early warning effectiveness. Furthermore, the viral sequence most closely related to the virus infecting the case was identified as submitted from Taiwan, China. A total of three close contacts of the patient were identified; none of whom exhibited typical clinical symptoms, such as fever or rash. However, one tested positive for mpox IgG antibodies, suggesting that the Taiwan lineage virus has established silent transmission chains within the local high-risk population network associated with the case; these chains were not detected via routine surveillance.

Currently, most surveillance and epidemiological investigations primarily rely on nucleic acid testing, without dynamic serological monitoring, making it difficult to systematically identify past infections, silent exposures, and complete transmission chains. Serological testing serves as a key tool for identifying silent exposure, reconstructing transmission chains, and clarifying the chronology of infection (18). It can also effectively detect past exposure, asymptomatic infection, and silent transmission. Notably, a key advantage of serological surveillance is its extended detection window. Infected individuals may present without a typical rash or with only mild symptoms and may test negative for viral nucleic acid; however, IgM and IgG antibodies may persist for extended periods, enabling the identification of cases missed by nucleic acid-based screening alone. This capability addresses the critical limitation of the current surveillance system of exclusively relying on case reports and nucleic acid screening. In low-incidence regions, conducting serological antibody screening can help assess the actual exposure pressure among high-risk populations, determine the presence of local hidden transmission cycles, and inform prevention and control strategies. Our present study suggests that incorporating IgM and IgG antibody testing into close contact management and contact tracing can aid in effectively overcoming the limitations of nucleic acid testing, thereby enhancing the accuracy and completeness of transmission chain identification and providing important evidence for refining mpox epidemiological investigation protocols. However, a positive result for mpox IgM or IgG antibodies does not directly indicate current infection. When surgical data are used to determine active infection or define cases, a strategy involving multiple quality control measures and combined interpretation is crucial to avoid misclassification caused by false positives.

This study has certain limitations that warrant cautious interpretation of the findings. First, this study included only one confirmed case and their close contacts. Therefore, the findings lack representativeness and cannot fully reflect the overall epidemiological characteristics of co-infection with mpox and HIV in regions with low incidence rates or the general patterns of asymptomatic transmission. Second, data completeness was compromised by the inherent limitations of the retrospective study design. Specifically, recall bias may have affected the accuracy of the epidemiological information. Furthermore, the absence of viral genome sequencing of mpox close contacts and environmental sampling precludes the definitive identification of the source of transmission or assessment of the risk of environmental transmission. Third, serological evidence was limited to a single IgG antibody test, which indicates past exposure but cannot determine when exposure occurred. As no dynamic serological surveillance system has been established, the time of exposure, the sequence of infection, or links within transmission chains could not be determined based on changes in antibody titers. Consequently, contact tracing efforts could not be pursed in depth; the timing of exposure, stage of infection, and infectiousness of individuals remain unclear, limiting the ability to draw definitive conclusions regarding transmission.

## 5 Conclusion

With this study, we highlight the importance of prioritizing the assessment of the risk of asymptomatic transmission in mpox prevention and control strategies in low-incidence areas. This can be achieved by strengthening active screening of high-risk groups, reducing over-reliance on healthcare-seeking behavior, and establishing and refining dynamic serological surveillance systems and mechanisms to enhance early identification and control of asymptomatic transmission. Future research should involve larger, multicenter prospective studies integrating genomic sequencing with longitudinal serological surveillance. Such studies would help validate the intensity of local transmission, clarify the epidemiological characteristics of asymptomatic transmission, optimize mpox prevention and control strategies in low-incidence areas, and address current gaps in relevant research.

## Data Availability

The raw data supporting the conclusions of this article will be made available by the authors, without undue reservation.
